# Effects of Refined Xiaoyaosan on Depressive-Like Behaviors in Rats with Chronic Unpredictable Mild Stress through Neurosteroids, Their Synthesis and Metabolic Enzymes

**DOI:** 10.3390/molecules22081386

**Published:** 2017-08-21

**Authors:** Xiaoling Guo, Wenqi Qiu, Yueyun Liu, Yifang Zhang, Hongbo Zhao, Jiaxu Chen

**Affiliations:** 1 School of Basic Medical Science, Beijing University of Chinese Medicine, Beijing 100029, China; guoxiaoling.tcm@163.com (X.G.); qiuwq@bucm.edu.cn (W.Q.); chloelou@126.com (Y.L.); zhangyifang0625@163.com (Y.Z.); zhaohongbo2007@sina.com (H.Z.); 2 Beijing Fengtai District Fangzhuang Community Health Service Center, Beijing 100078, China

**Keywords:** refined XYS, depression, rats, behavior, plasma, hippocampus, amygdala, neurosteroids, synthesis and metabolic enzyme, mRNA expression

## Abstract

To observe the effects of refined Xiaoyaosan (XYS) on the depressive-like behaviors in rats with chronic unpredictable mild stress (CUMS), and to explore the relationship between the changes of neurosteroids and mRNA expressions of their synthesis and metabolic enzymes, and the mechanism of XYS in the treatment of depression. *Methods*: Eighty-four healthy male Sprague-Dawley rats were randomly divided into normal group, model group, XYS group and fluoxetine group. The latter three groups were subjected to 21 days of CUMS to prepare the stress depression model. Rats in the XYS group, and fluoxetine group were given intragastric administration with refined XYS and fluoxetine, respectively. The behavioral changes of the rats were observed after 21 days. The contents of pregnenolone (PREG), progesterone (PROG) and alloprognanolone (ALLO) in the plasma of rats were measured by ELISA. The levels of PREG, PROG and ALLO in the hippocampus and amygdala tissues were measured by LC-MS/MS. The mRNA expressions of 3α-hydroxysteroid dehydrogenase (3α-HSD), 3β-hydroxysteroid dehydrogenase (3β-HSD), cholesterol side-chain cleavage enzyme (P450scc) and 5α-reductase (5a-R) in the hippocampus and amygdala were detected by RT-qPCR methods. *Results*: There were changes in the model rats. The contents of PREG, PROG and ALLO changed similarly, which reflected in the decrease of PROG and ALLO, and the increase of PREG. The mRNA expression of P450scc was increased, and the mRNA expressions of 3α-HSD, 3β-HSD and 5a-R were decreased. Refined XYS could improve the behaviors of rats and the biological indicators. *Conclusions*: There is a neurosteroid dysfunction in the brain region of depression rat model animals, and the mechanism of refined XYS depression treatment may be related to the regulation of the control of mRNA expression of related synthesis and metabolic enzymes in the hippocampus and amygdala, further affecting the contents of neurosteroids.

## 1. Introduction

Depression is a common emotional disorder disease, in which patients have no interest in anything, persistent mood depression, and associated symptoms such as anxiety, physical discomfort, loss of appetite, slow response, and suicidal thoughts [1,2]. With the accelerated pace of life, the incidence of depression, as one of the major diseases that endanger human mental health, has increased year by year. The morbidity of China has reached 11.7% [3], but its pathogenesis is not yet fully clear. The hypothesis that depression is due to a decrease of monoamine neurotransmitter is the most prevalent theory, but the drugs targeting the regulation of monoamine neurotransmitter can only alleviate symptoms of some patients [4], indicating that is a decrease of monoamine neurotransmitter is not the only pathogenesis of depression, but also the plasticity disorder of the related neural circuity plays a key role [5]. The hippocampus and amygdala are the two most important brain regions in the study of depression. The two are not only the important components of the emotional center, but also the key sites of depression caused by the uncontrolled HPA axis negative feedback and over-activation induced by chronic stress [6,7]. Recent studies have shown that there are abnormal changes of morphology and chemical composition in the limbic system of patients with depression [8,9].

Neurosteroids is the general term for steroids which have been found in recent years that regulate the excitability of neurons by neurotransmitters, which are synthesized by the central nervous system, or synthesized by the peripheral nervous system but metabolized by the central nervous system. Most neurosteroids regulate the neurotransmission of various neurotransmitters, and regulate behavior such as anxiety, depression, and eating, which depend on the types of neurosteroids, the brain region, and the presynaptic membrane function [10]. Current studies have shown that severe depression or chronic stress can lead to a reduction in the majority of neurosteroids in the central nervous system [11], suggesting that neurosteroids and their rate-limiting enzymes may be potential antidepressant targets. Neurosteroids can rapidly regulate the nerve excitability through inhibitory GABA type A receptors (GABAARs) and/or N-methyl-D-aspartate receptors (NMDARs) [12], such as ALLO can enhance the response of GABAAR, PREGS and DHEA enhance NMDAR function and attenuate GABAAR function [13].

Cholesterol is the precursor of all neurosteroids, and it can be called the ancestor of neurosteroids. Under the action of P450scc in the mitochondrial inner membrane, cholesterol generates PREG, which generates more neurosteroids. PREG is derivatized to PROG catalyzed by 3β-HSD. Under the action of 5α-R, PROG generates 5α-dihydroprogesterone, which can further generate ALLO. PROG can also metabolize to generate tetrahydrodeoxycorticosterone, allopregnanolone and other neurosteroids. In this whole process, some important neurosteroids, as well as the related key synthesis and metabolic enzymes were selected as the indicators of the experiment.

XYS was first described in the “Taiping Huimin Heji Jufang”, and it was used to treat emotional illness. Our previous experimental study [14] and a large number of clinical reports [15] show that XYS, a prescription for dispersing stagnated liver qi to relieve qi stagnation [16], and invigorating spleen and nourishing blood, has a good effect on depression. Therefore, in order to further explore the antidepressant mechanism of XYS, we chose the neurosteroids and related enzymes to study the changes in depression rats and the effect of XYS. Fluoxetine, which is a selective serotonin reuptake inhibitor (SSRI) type of antidepressant, was used as a positive control drug. In this study, we used CUMS to establish a rat model of depression, evaluated by sugar consumption, novel inhibition of feeding and open field behavior test to observe the effects of refined XYS on rats’ behaviors, the levels of neurosteroids in plasma, hippocampus and amygdala, and the mRNA expressions of their synthesis and metabolic enzymes.

## 2. Results

### 2.1. Effect of XYS on Body Weight of CUMS Rats

**Figure 1:**
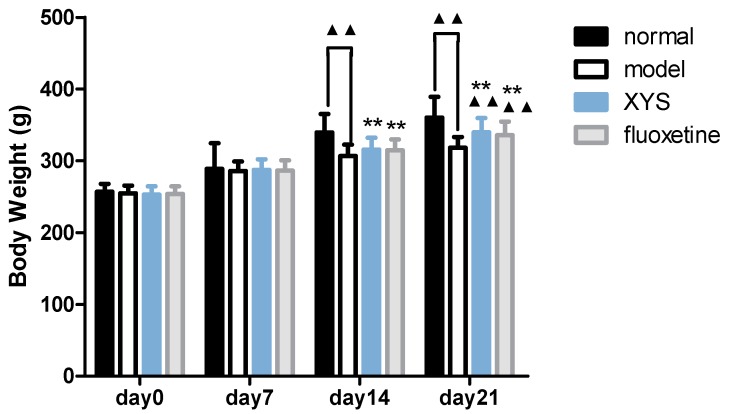
Body weight of each group. The data were expressed as mean ± standard deviation ($\bar{x}$ ± s), *n* = 21. ^▲▲^ *p* < 0.01, compared with the model group; ** *p* < 0.01, compared with the normal group.

Compared with the model group, the body weights (Figure 1) of the normal group, XYS group and fluoxetine group were increased on the 14th day, and there was significant difference between the normal group and the model group (^▲▲^ *p* < 0.01). Compared with the model group, the body weights of the normal group, XYS group and fluoxetine group were significantly increased on the 21st day (^▲▲^ *p* < 0.01).

### 2.2. Effect of XYS on Sugar Consumption Rate and Novelty Suppressed Feeding Test of CUMS Rats

**Figure 2:**
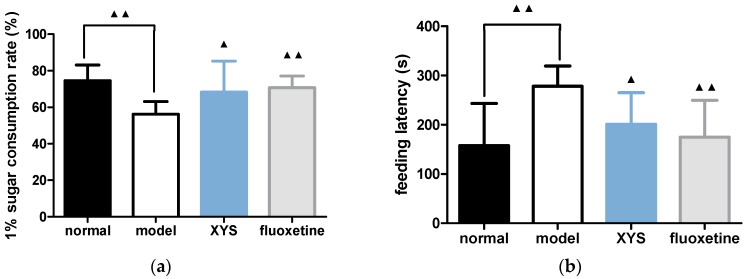
(**a**) Sugar consumption rate; (**b**) feeding latency. The data were expressed as mean ± standard deviation ($\bar{x}$ ± s), *n* = 10. ^▲^ *p* < 0.05, ^▲▲^ *p* < 0.01, compared with the model group.

Compared with the model group, the consumption of 1% sucrose water (Figure 2a) in the normal group, XYS group and fluoxetine group were higher than that in the model group, and the feeding latencies (Figure 2b) were lower (^▲^ *p* < 0.05, ^▲▲^ *p* < 0.01).

### 2.3. Effect of XYS on Open Field Test of CUMS Rats

**Figure 3:**
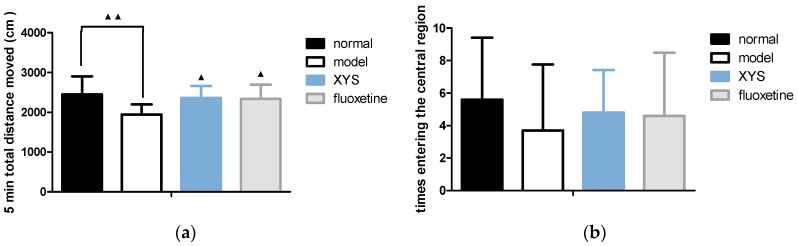

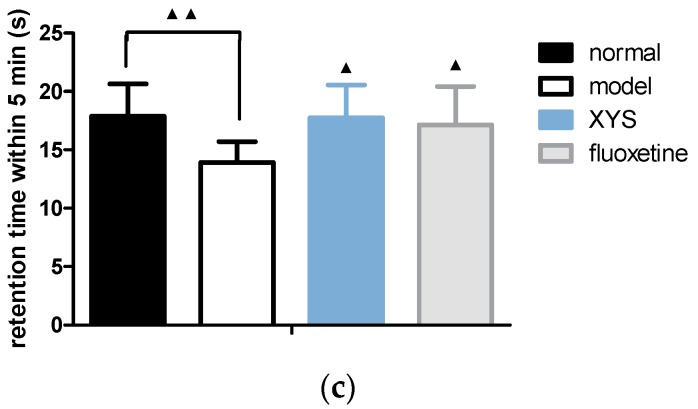
(**a**) Total distance moved; (**b**) numbers of rats entering the central area; (**c**) retention time in the central area. The data were expressed as mean ± standard deviation ($\bar{x}$ ± s), *n* = 10. ^▲^ *p* < 0.05, ^▲▲^ *p* < 0.01, compared with the model group.

Compared with the model group, the total distance moved (Figure 3a) of the other three groups were longer, and the retention time in the central area (Figure 3c) were longer within 5 min (^▲^ *p* < 0.05, ^▲▲^ *p* < 0.01). The numbers of rats entering the central area (Figure 3b) were increased, but there was no significant difference.

### 2.4. Effect of XYS on the Contents of PREG, PROG and ALLO in Plasma of CUMS Rats

**Figure 4:**
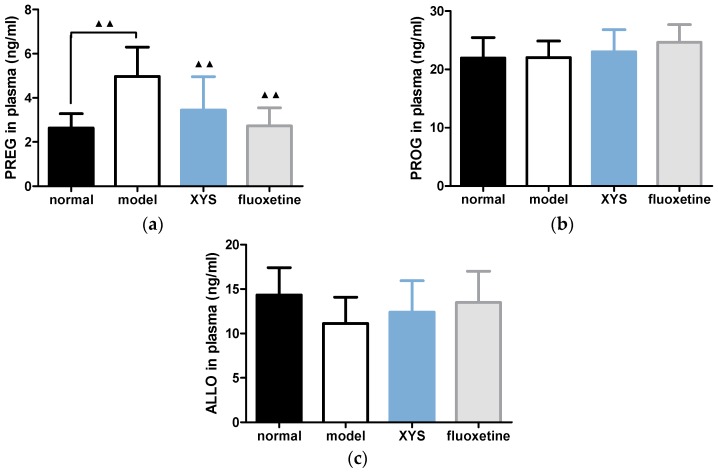
(**a**) Plasma PREG content; (**b**) plasma PROG; (**c**) plasma ALLO. The data were expressed as mean ± standard deviation ($\bar{x}$ ± s), *n* = 10. ^▲▲^ *p* < 0.01, compared with the model group.

Compared with the model group, the contents of PREG in the other three groups (Figure 4a) were decreased (^▲▲^ *p* < 0.01). The content of PROG in the normal group (Figure 4b) was decreased slightly, and the contents of PROG in the XYS and fluoxetine groups was increased slightly, but there was no significant difference. The contents of ALLO in other three groups were increased (Figure 4c), but there was no significant difference.

### 2.5. Effect of XYS on the Contents of PREG, PROG and ALLO in Brain of CUMS Rats

#### 2.5.1. Effect of XYS on the Contents of PREG, PROG and ALLO in Hippocampus of CUMS Rats

**Figure 5:**
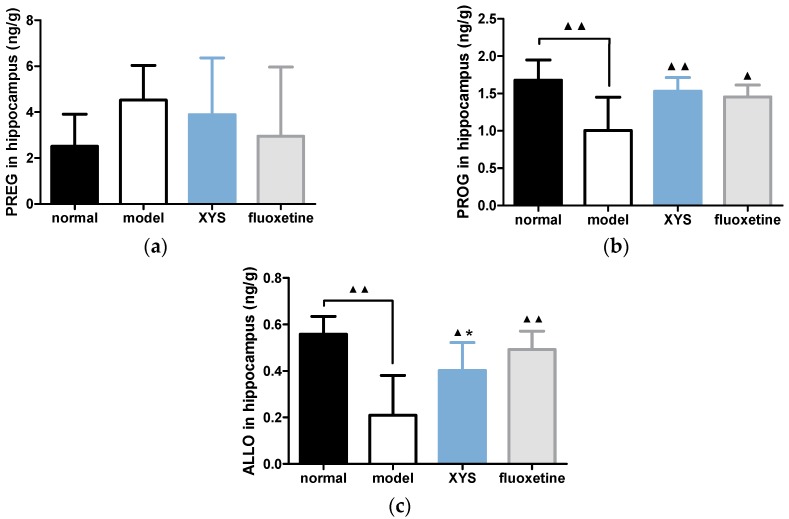
(**a**) PREG content; (**b**) PROG content; (**c**) ALLO content in hippocampus. The data were expressed as mean ± standard deviation ($\bar{x}$ ± s), *n* = 10. ^▲^ *p* < 0.05, ^▲▲^ *p* < 0.01, compared with the model group; * *p* < 0.05, compared with the normal group.

Compared with the model group, the contents of PREG (Figure 5a) in other three groups were decreased (*p* > 0.05), but there was no significant difference. The contents of PROG (Figure 5b) and ALLO (Figure 5c) in other three groups were significantly higher than that in the model group (^▲^ *p* < 0.05, ^▲▲^ *p* < 0.01).

#### 2.5.2. Effect of XYS on the Contents of PREG, PROG and ALLO in Amygdala of CUMS Rats

**Figure 6:**
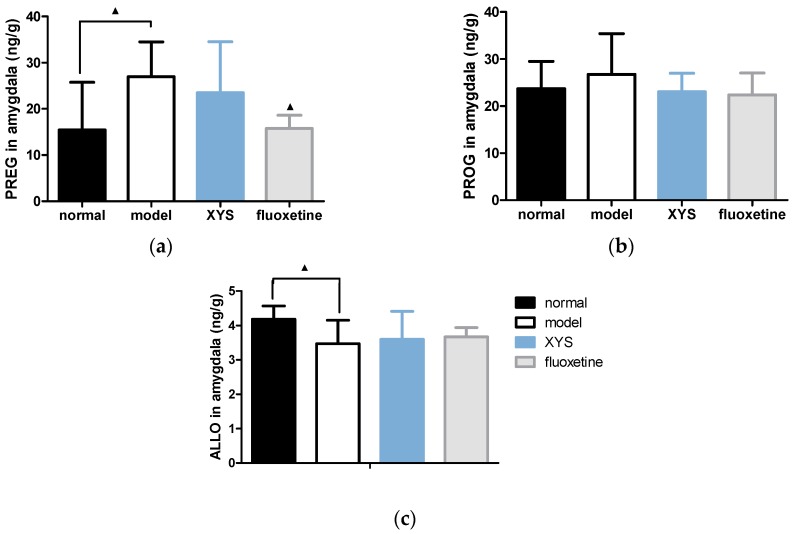
(**a**) PREG content; (**b**) PROG content; (**c**) ALLO content in the amygdala. The data were expressed as mean ± standard deviation ($\bar{x}$ ± s), *n* = 10. ^▲^ *p* < 0.05, compared with the model group.

Compared with the model group, the contents of PREG in the other three groups were decreased (Figure 6a), and there were significant differences in normal group and fluoxetine group (^▲^ *p* < 0.05). The contents of PROG (Figure 6b) in other three groups were decreased, but there was no significant difference. The contents of ALLO (Figure 6c) in other three groups were increased, and there was significant difference in the normal group (^▲^ *p* < 0.05).

### 2.6. Effect of XYS on the mRNA Expressions of 3α-HSD, 3β-HSD, P450scc and 5α-R of CUMS Rats

#### 2.6.1. Effect of XYS on the mRNA Expressions of 3α-HSD, 3β-HSD, P450scc and 5a-R in Hippocampus of CUMS Rats

**Figure 7:**
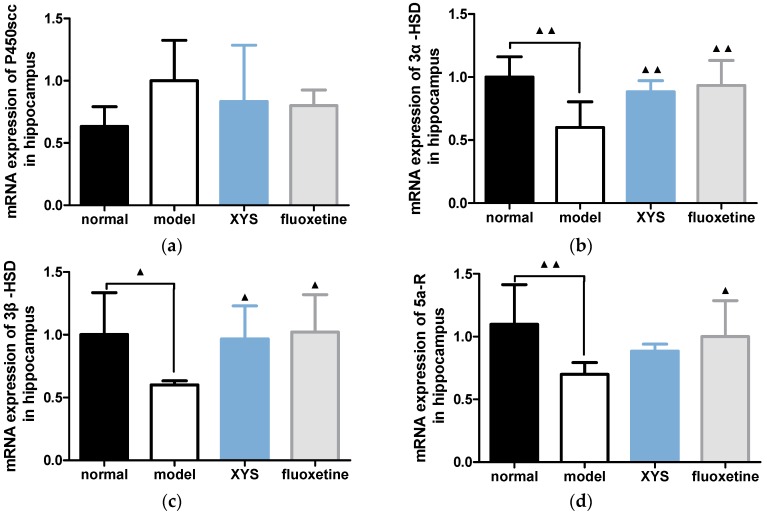
(**a**) mRNA expression of P450scc in hippocampus; (**b**) 3a-HSD; (**c**) 3β-HSD; (**d**) 5a-R. The data were expressed as mean ± standard deviation ($\bar{x}$ ± s), *n* = 10. ^▲^ *p* < 0.05, ^▲▲^ *p* < 0.01, compared with the model group.

Compared with the model group, the mRNA expressions of P450scc in other three groups were decreased (Figure 7a), but there was no significant difference. The mRNA expressions of 3α-HSD (Figure 7b) and 3β-HSD (Figure 7c) in the other three groups were all statistically increased (^▲^ *p* < 0.01, ^▲▲^ *p* < 0.05). The mRNA expression of 5a-R (Figure 7d) in the other three groups were increased (^▲^ *p* < 0.01, ^▲▲^ *p* < 0.05).

#### 2.6.2. Effect of XYS on the mRNA Expressions of 3α-HSD, 3β-HSD, P450scc and 5a-R in Amygdala of CUMS Rats

**Figure 8:**
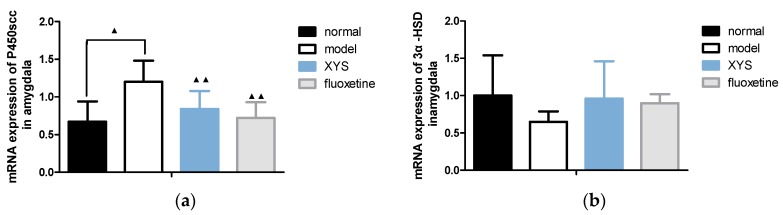

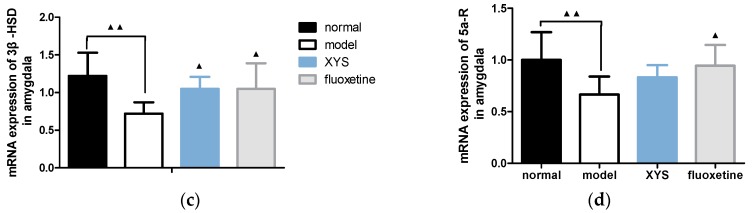
(**a**) mRNA expression of P450scc in amygdala; (**b**) 3a-HSD; (**c**) 3β-HSD; (**d**) 5a-R. The data were expressed as mean ± standard deviation ($\bar{x}$ ± s), *n* = 10. ^▲^ *p* < 0.05, ^▲▲^ *p* < 0.01, compared with the model group.

Compared with the model group, the mRNA expressions of P450scc (Figure 8a) and 5a-R (Figure 8d) in the other three groups were decreased (^▲▲^ *p* < 0.01, ^▲^ *p* < 0.05). The mRNA expression of 3α-HSD (Figure 8b) in the other three groups were slightly higher (*p* > 0.05). The mRNA expression of 3β-HSD (Figure 8c) in the other three groups were increased (^▲▲^ *p* < 0.01, ^▲^ *p* < 0.05).

## 3. Discussion

In this study, an internationally accepted CUMS method [17,18] was used to establish a rat depression model. The model was evaluated by body weight, sugar consumption rate, novelty suppressed test and open field test, which confirmed that the model basically simulated depressive effects such as the lack of pleasure, lack of interest, mental retardation and decreased body weight, suggesting that the model was successfully implemented. At the same time, the evaluations of the XYS group and fluoxetine group were statistically significant compared with the model group, suggesting that refined XYS and fluoxetine could significantly improve the behaviors of depressed rats.

Chronic stress affects the reception and digestion of the spleen and stomach, and loss of appetite, reduced food intake, slow weight growth and other symptoms will appear, indicating that rats cannot self-adjust under excessive stress [19,20]. After the treatment with XYS, the weight loss trend was curbed. Rodents prefer sweet diets, and reduced preference for sugar consumption means a lack of pleasure in depression, so sugar consumption can detect the emotional state or sensation of rodents [21]. 1% sucrose water consumption rates showed a statistical difference, and XYS can effectively improve this indicator. In the novelty suppressed test, the length of feeding latency can reflect the degree of anxiety/depression in rats. Decreased activity of rats in the open field test indicates that sustained stress leads to the obvious depressive states of rats such as reduced desire to explore the surrounding environment, physical and psychological frustration [22].

In recent years, neural plasticity has been a hot spot in the pathogenesis of depression, especially morphological and functional plasticity. The hippocampus and amygdala are the two most important brain regions in depression, and the hippocampus is mainly associated with learning and memory, while the amygdala is the main brain region that mediates aversion. Recent studies have shown that the limbic system of patients with depression has changed abnormally in both morphological and chemical composition. For example, the chronic immobilization stress depression model can decrease the positive expression of AMPA receptor GluR2/3 subunit in the hippocampal CA1 region and increase the positive expression in basolateral amygdala [23]. Another report shows that there is a decrease in hippocampus and cause an increase in the synaptic plasticity of the amygdala [15]. The above show that the imbalance of hippocampus-amygdala nerve plasticity is an important pathological mechanism of depression.

Severe depression or chronic stress can lead to the reduction of most neurosteroids in the central nervous system [11]. Antidepressants, while improving depressive symptoms, also recover the neurosteroid levels. Systemic/central administration of neurosteroids such as ALLO, DHEA, E2, etc. can reduce depression-like behaviors, and rate-limiting neurosteroid enzymes such as 5α-reductase, 3α-hydroxysteroid dehydrogenase also undergo similar changes [24]. These reports have shown that neurosteroids play an important role in the pathophysiology process of depression, suggesting that neurosteroids and their rate-limiting enzymes may be potential antidepressant targets. In our previous study, the protein expression of P450scc in the hippocampus of depressed rats was significantly decreased, and XYS could reverse it effectively [25]. However, the expression of P450scc shows opposite changes in the transcriptome and proteome, which needs further study of the process of post-translational modification.

This study showed that the changes of PREG, PROG and ALLO in plasma, hippocampus and amygdala of depression rats induced by CUMS were similar. PREG increased in amygdala and plasma and showed an increasing trend in hippocampus. ALLO decreased in amygdala and hippocampus and showed a decreasing tendency in plasma. PROG decreased in hippocampus and showed a tendency to increase in amygdala. The mRNA expressions of 3α-HSD, 3β-HSD and 5a-R in hippocampus and amygdala of rats were all decreased and the expression of P450scc was increased. XYS and fluoxetine can effectively reverse the above changes. According to the preceding discussion, we have learned and confirmed that there exist imbalances of neural plasticity in rats with depression. A large number of studies have confirmed that the level of γ-aminobutyric acid (GABA) [26,27] in patients with depression is abnormally low, so that GABA also has a relationship with antidepressants. The neurosteroids PROG and ALLO are GABA agonists, and can enhance the inhibitory effect of GABA, enhance the NMDA receptors, but inhibit the release of glutamate. PREG and its sulfate are both GABA inhibitors. This study concluded that PROG and ALLO in plasma, hippocampus and amygdala were decreased, while PREG was increased, and XYS could effectively reverse the above changes. It is thus speculated that XYS plays a role in the treatment of depression by increasing PROG and ALLO and reducing PREG in hippocampus and amygdala, thus enhancing the release of GABA and NMDA receptors, inhibiting the release of glutamate receptors and reducing the inhibitory effect of GABA.

## 4. Materials and Methods

### 4.1. Animals and Grouping

Healthy male Sprague-Dawley (SD) rats, with age of 8–10 weeks and bodyweight of (180 ± 10) g, were provided by Beijing Weitong Lihua Experimental Animal Center (number 2006-000, Beijing, China). All animals were habituated for one week before performing further experiment, with five rats per cage. All animals were fed in standard animal room (room temperature: 22 ± 2 °C; relative humidity: 30–40%; keeping the animal room quiet). Regular pellet food and purified water were available ad libitum. 84 SD rats were randomly divided into four groups: normal group, model group, XYS group and fluoxetine group, with 21 rats in each group.

### 4.2. Drug Preparation and Intervention

The herbal formula of XYS comprises the following drugs: *Angelicae sinensis* Radix (root of *Angelica sinensis* (Oliv.)Diels), Paeoniae Radix Alba (root of *Paeonia lactiflora* Pall.), Bupleuri Radix (root of *Bupleurum chinenese* DC), Atractylodis Macrocephalae Rhizoma (root and rhizome of *Atractylodes macrocephala* Koidz), Glycyrrhizae Radix et Rhizoma (root and rhizome of *Glycyrrhiza uralensis* Fish), Poria (fungus nucleus of *Poria cocos* (Schw.) Wolf), Zingiberis Rhizoma Recens (root and rhizome of *Zingiber officinale* Rosc.), and Menthae Haplocalycis Herba (overground parts of *Mentha haplocalyx* Briq.) in a ratio of 6:6:6:6:6:2:2:3. The traditional Chinese medicine materials used in this study were provided by Beijing Tongrentang (Bozhou) Pieces Co., Ltd. (Bozhou, China), adopting the standard “Beijing Chinese Medicine Processing Standard” [28,29,30] which had been widely used. According to the proportion of the original recipe and adopting the extraction method of traditional Chinese medicine compound, refined XYS (including powder and volatile oil) was prepared, and it was formulated to a certain concentration according to need. Since the first day of modeling, rats were given intragastric administration for 21 days 1 h before modeling. XYS group was given refined XYS (powder 0.0197 g/100 g, volatile oil 0.0032 mL/100 g, according to the average adult body weight 60 kg/d dose conversion). The fluoxetine group was given fluoxetine suspension, at a dose of 0.2 mg/100 g body weight. The normal group and model group were given the same amount of solvent (0.5% *w*/*v* Tween 80 dissolved in 1% CMC-Na) [31], which refined XYS and fluoxetine were prepared with the solvent.

### 4.3. CUMS Model Preparation

The normal group was fed (water and food) normally, without any stimulation. The other three groups received a variety of stress stimulation for 21 days. According to Katz and others [32], the stimulation method included clipping the tail for 1 min (1 cm from the tail root), fasting for 24 h, water-deprivation for 24 h, ice water swimming (4 °C, 5 min), noise for 1 h (1500 Hz, 92 dB), behavior restriction for 3 h, hot baking (45 °C, 5 min), humid environment for 24 h, social isolation, day and night reversed and other stimuli. Stimulations were applied randomly 1–2 times a day at different times during 21 days. The same kind of stimulation is not used continuously, so that animals cannot predict the occurrence of stimulation.

### 4.4. Body Weight, Sugar Consumption Rate, Novelty Suppressed Feeding Test, Open Field Test

At the beginning of the experiment, the body weight of the rats was weighed by an electronic scale every 3 days. The dose was adjusted according to the body weight, and the body weight of the rats on the 0th, 7th, 14th and 21st day were compared.

The sugar consumption rate was measured at the end of 21 d modeling according to [33]. Each rat was kept in separation. Rats were firstly trained for 72 h, with two bottles of 1% sugar during the first 24 h, one bottle of 1% sugar and one bottle of pure water during the second 24 h and fasting during the third 24 h. Then at the same time, each rat was given two bottles (1% sugar and pure water, 100 mL). Consumptions of pure water and 1% sugar within 1 h were measured to calculate the consumption rate of sugar. Sugar consumption rate (%) = sugar consumption (mL)/[sugar consumption (mL) + pure water consumption (mL)].

Novelty suppressed feeding test was using a white square open box (50 cm × 50 cm × 40 cm), with 2 cm wooden bedding placed on the floor. 30 pellets of food (regular chow) were placed in the center. After food deprivation for 48 h, rats were individually placed in the same corner of the open box. The latency to begin chewing the pellets (rats began to bite the pellets rather than only sniff or play pellets) was recorded for up to 5 min. White paper and food were replaced for every rat. The feeding latency and the length of eating time were all affected by anxiety/depression in rats [34].

The open field test began at 8:00 a.m. on day 22 of the modeling, and was improved by the literature [35]. The activity of the rats was measured in a 100 cm × 100 cm × 40 cm cube with wood walls and wood floor, without ceiling (handmade), which is covered by gray paint. The chambers were individually divided into 25 squares by yellow paint. The activity monitor camera was on the top of the middle square, and both horizontal and vertical movements were analyzed by Observer 5.0 software (Noldus, Wageningen, The Netherlands) and EthoVision 3.0 software (Noldus). Each rat was placed in the central square and observed for 5 min. The total distance moved, the times of rats entering the central region and the retention time in the central region within 5 min were calculated.

### 4.5. ELISA Analysis

The contents of plasma PREG, PROG and ALLO were measured by ELISA assays. After the rat behavioral test, 10 rats in each group were given 10% chloral hydrate intraperitoneal anesthesia (0.35~0.40 mL/100 g body weight). 2 mL abdominal aorta blood was collected and centrifuged 20 min (3500 r, 4 °C). ELISA assays of PREG, PROG, ALLO were performed according to the kit instructions (PROG was detected by a competition method, while PREG and ALLO were detected by a double antibody sandwich method).

### 4.6. LC-MS/MS Analysis

The contents of PREG, PROG and ALLO in the hippocampus and amygdala were detected by LC-MS/MS. After the rat behavioral test, six rats in each group were intraperitoneally anesthetized with 10% chloral hydrate, and the bilateral hippocampus and amygdala were quickly removed and added into 1.5 mL RNase-Free EP tube, and stored at −80 °C. Chromatographic separation was performed using an Agela MPC18 column (100 × 2.1 mm, 5 μm), Lot No: VA950502-0. The chromatographic separation conditions were: mobile phase A: aqueous solution, B: acetonitrile; flow rate: 1 mL/min (gradient elution); injection volume: 10 μL; column temperature: 25 °C. Mass spectrometry conditions: ion source for Turbo Ionspray (APCI); positive ion ionization mode; scanning method: multiple reaction monitoring (MRM); ion source voltage 4500 V; ion source temperature 550 °C. The MRM settings of PREG, PROG, ALLO and the internal standard (methyl testosterone) were *m*/*z* 493.4→152.0, *m*/*z* 495.4→152.0, *m*/*z* 497.5→152.0 and *m*/*z* 481.6→152.0 respectively.

### 4.7. RT-qPCR Analysis

After the rat behavioral test, six rats in each group were intraperitoneally anesthetized with 10% chloral hydrate, and the bilateral hippocampus and amygdala were quickly removed and added into 1.5 mL RNase-Free EP tube, and stored at −80 °C. The total RNA of tissue was extracted using a mirVana™ miRNA Isolation Kit (ABI, Foster City, CA, USA). The mRNA expressions of 3α-HSD, 3β-HSD, P450scc and 5α-R in the hippocampus and amygdala were detected by the RT-qPCR method. The cycle threshold (*C*_t_) was detected by monitoring the changes in the fluorescence intensity signal using fluorescence quantitative PCR instrument (ABI). The amounts of 2× SYBGREEN PCR master mix, reverse transcription product of cDNA, upstream primer, downstream primer and PCR H_2_O were 15, 1, 0.5, 0.5 and 13 μL respectively. Reaction conditions: 95 °C pre-denaturation 10 min, 95 °C denaturation 15 s, 60 °C annealing 30 s, 72 °C amplification 30 s, a total of 40 cycles. According to the Genebank sequence, each primer sequence was designed and synthesized. In this study, β-actin was used as the internal reference gene, and the quantitative analysis of fluorescence PCR results was carried out by 2^−^^△△*C*t^ method.

### 4.8. Statistical Methods

The measurement data were expressed as mean ± standard deviation ($\bar{x}$ ± s). SPSS 17.0 software was used for statistical analysis, using one-way ANOVA and repeated measures ANOVA. SNK method was used to compare the data between groups. *p* < 0.05 for the difference was statistically significant.

## 5. Conclusions

Changes of PREG, PROG and ALLO levels in the hippocampus and amygdala, and mRNA expression changes of their synthesis and metabolic enzymes 3α-HSD, 3β-HSD, P450scc and 5α-R are the intrinsic biological basis of stress depression and the endocrine mechanism of the central nervous system, and this study initially reveals that XYS may play a role in the treatment of stress depression through the regulation of neurosteroids in the hippocampus and amygdala. Neurosteroids may be one of the targets, and this study enriches the scientific knowledge of the basis of the effect of XYS in the treatment of stress-induced depression.
